# Bumblebee Homing: The Fine Structure of Head Turning Movements

**DOI:** 10.1371/journal.pone.0135020

**Published:** 2015-09-09

**Authors:** Norbert Boeddeker, Marcel Mertes, Laura Dittmar, Martin Egelhaaf

**Affiliations:** 1 Department of Neurobiology & Center of Excellence ‘Cognitive Interaction Technology’ (CITEC), Bielefeld University, Bielefeld, Germany; 2 Department of Cognitive Neurosciences & Center of Excellence ‘Cognitive Interaction Technology’ (CITEC), Bielefeld University, Bielefeld, Germany; University of Muenster, GERMANY

## Abstract

Changes in flight direction in flying insects are largely due to roll, yaw and pitch rotations of their body. Head orientation is stabilized for most of the time by counter rotation. Here, we use high-speed video to analyse head- and body-movements of the bumblebee *Bombus terrestris* while approaching and departing from a food source located between three landmarks in an indoor flight-arena. The flight paths consist of almost straight flight segments that are interspersed with rapid turns. These short and fast yaw turns (“saccades”) are usually accompanied by even faster head yaw turns that change gaze direction. Since a large part of image rotation is thereby reduced to brief instants of time, this behavioural pattern facilitates depth perception from visual motion parallax during the intersaccadic intervals. The detailed analysis of the fine structure of the bees’ head turning movements shows that the time course of single head saccades is very stereotypical. We find a consistent relationship between the duration, peak velocity and amplitude of saccadic head movements, which in its main characteristics resembles the so-called "saccadic main sequence" in humans. The fact that bumblebee head saccades are highly stereotyped as in humans, may hint at a common principle, where fast and precise motor control is used to reliably reduce the time during which the retinal images moves.

## Introduction

Salient objects can help insects like bees, wasps and ants to accurately find their way back home and to newly discovered food sources [1–2]. In experiments that are designed to find out *what* features of the environment the homing insects actually use, the experimenters often allow the animals to become accustomed to distinct visual features close to the place of interest, i.e. their feeding site, and then displace or modify these landmark cues with the aim of observing where and how the animals search for the goal. From such experiments it is clear that insects can use the retinal size and position, the colour, distance and texture of landmarks, [3–8] as well as skyline elevations for homing [9]. Recent experiments with landmarks that were camouflaged by carrying the same texture as the background, suggest that honeybees can also exploit dynamic cues like the optic flow pattern to pinpoint the goal location [10].

To understand *how* insects find home, i.e. to unravel the mechanisms they use to extract spatial information from their retinal input, it is crucial to analyse the temporal organization of their behaviour [11]. Bumblebees like honeybees and wasps have evolved highly structured flight patterns for place learning, often called learning or turn-back-and-look-flights [12–13]. They perform turn-back-and-look (TBL) flights when leaving their nest for the first time or when leaving a newly discovered food source. These learning routines are crucial for subsequent successful homing (reviewed in [14]) and indicate an active vision strategy that helps bees to navigate by utilizing translational optic flow [10]. The specific pattern of optic flow is determined by both the layout of the environment and by the animal’s behaviour [15–16]. Depending on their flight style bees, like other flying animals, can experience two basic types of image motion patterns, one is due to rotations of the eyes (rotational optic flow), and one is due to translatory motion (translational optic flow). The rotational optic flow component is generated by orientation changes of the eye; image displacements have uniform directions across the visual field and amplitudes are independent of the distance to objects and depend on the dynamics of the eye rotation. In contrast, optic flow generated by translation depends on egomotion parameters and on the distance of objects in the world. Translational flow thus contains range information as images of close objects move faster across the retina than those of more distant objects [17].

Head and eye movements can shape and reduce the complexity of optic flow creating favourable conditions for image analysis [18–19]. During view-based homing the bees' flight style facilitates depth perception from motion parallax [10; 20–21]: the bees’ trajectories consist of straight flight segments combined with rapid turns about the vertical body axis. Because bees and other insects cannot move their eyes relative to the head capsule, the direction of gaze is determined by the orientation of the head. By analogy with human eye movements, these rapid changes of gaze direction have been called saccades [22]. Between saccades gaze direction is mostly constant since stabilizing head movements largely cancel out rotational optic flow around all three rotational axes perceived during free flight. Hence, head orientation is stabilized for most of the time except for fast changes in the horizontal gaze direction that serve to compress the visual system’s exposure to rotational optic flow into very brief moments in time. Gaze changes involve coordinated head- and body movements, whereby head saccades are faster and shorter than body [23–24]. A seminal study shows how precisely blowflies compensate rotations of the thorax in flight by counter rotations of the head relative to the [25–26]. In locusts and blowflies it has been shown that the processing of depth information from motion parallax crucially depends on a precise gaze stabilisation against rotations [27–28]

In the past years, research on learning, memory and visual navigation has increasingly focussed on bumblebees. One important reason for using bumblebees as a model animal is the possibility to house them indoors, which allows experiments throughout the year [29]. Furthermore, bumblebees are more robust in comparison with honeybees, which makes them suitable for several neurobiological approaches like calcium imaging [30] or single cell intracellular recording [31–34]. Additionally, bumblebees show very similar learning abilities like honeybees, e.g. in pattern discrimination experiments [35–36], or conditioning studies exploiting the proboscis extension response [37]. But in particular, they are exquisitely capable of solving navigational tasks [23;38]. To understand *how* bumblebees find home and to unravel the behavioural mechanisms with which they extract spatial information we analyse the fine temporal organization of their behaviour and especially the structure of horizontal head and body turns during navigation. We recorded high-speed videos of eye- and body-movements of bumblebees during a homing task to investigate how they shift gaze during their TBL and return flights and discuss the impact of structured gaze movements on visual motion processing.

## Material and Methods

### General procedure and Experimental setup

We obtained commercial bumblebee hives from Koppert (Berkel en Rodenrijs, The Netherlands). The bumblebees where taken from their original carton box and then housed in custom-built Perspex boxes (300mm * 200mm * 300mm) that were connected to the experimental apparatus (see below) by a plastic tube (length of 500 mm, diameter of 30mm). The whole setup, the training and the recording procedures were similar to those used in a study, where the performance of honeybees in locating the feeder was probed by targeted modifications of landmark texture and the landmark-feeder arrangement [10]. Here we used bumblebee workers for the experiments. They were trained to collect sugar solution from a transparent feeder in a circular flight arena (diameter of 1.95m). The sidewall of this flight arena was 500mm high and covered with the same red-white Gaussian-blurred random dot pattern as the arena floor. A dome of white cloth surrounded and covered the upper part of the flight arena to prevent the bees from seeing external visual cues. Indirect illumination was provided by eight Dedo-Lights (DLH4; 150W each) placed outside the cloth around the arena and by nine 50 W halogen lamps from above. All lights ran on DC power and were positioned symmetrically with respect to the arena centre.

**Fig 1:**
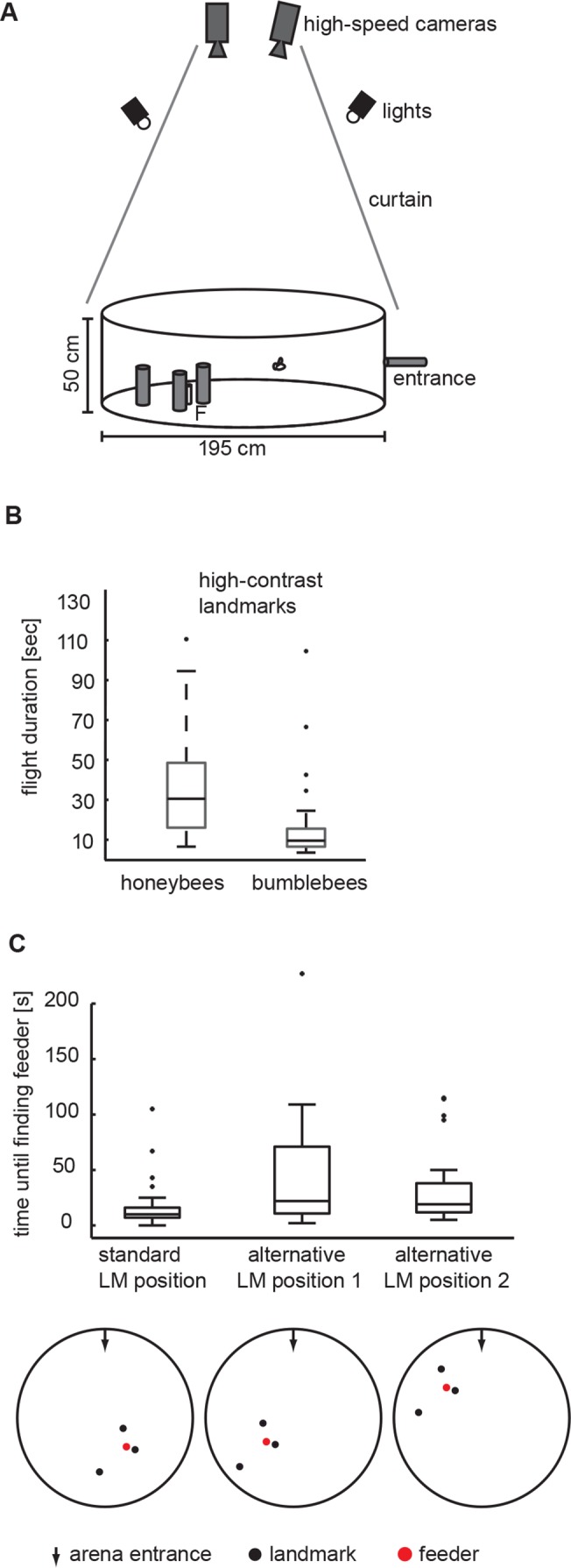
Flight arena and behavioural performance in the arena. (A) We trained honeybees and bumblebees to enter the arena via a hole in the sidewall. They were required to find a perspex feeder F between three landmarks placed around the feeder at different distances. Under indirect, uniform light conditions we recorded flight trajectories in the vicinity of the landmark arrangement using two high-speed cameras. (B) Boxplots of the time required for honeybees (n = 14; 68 flights) and bumblebees (n = 4; 33 flights) to land on the feeder. Time was taken when touching the feeder during landing. Box symbols: central horizontal line within the box–median; box edges represent 25^th^ and 75^th^ percentiles; whiskers–most extreme data points that are not outliers (> 75^th^ percentile +1.5*box size or < 25^th^ percentile– 1.5 * box size). (C) Bumblebee flight times when changing the position of the landmark arrangement within the arena, while maintaining the geometry of the landmark arrangement constant (n = 74 flights). Boxplot legend same as in B. Pictograms below the boxplot indicate the three different training positions of the landmark arrangement in the flight-arena. The arrow at the top of each pictogram denotes the entrance to the flight-arena. Search times for the two alternative locations are significantly different compared to the filming position (p = 0.02), The search time for the two alternative locations are not significantly different from each other, (p = 0.8).

Bees that continued to visit the feeder regularly were individually marked with acrylic paint on thorax and abdomen. These bees were then trained to associate the food reward with a constellation of three cylinders we will refer to as landmarks. Landmarks had a height of 250mm and a diameter of 50mm and were covered with homogeneously red paper. They were placed at different distances (100, 200, 400mm) around the feeder, at angles of 120° to each other with the feeder in their centre (Fig 1). A drop of sugar solution was provided on the feeder, which was made of an upright Perspex cylinder (100mm high, 20mm diameter) carrying a Perspex disc (5mm high, 40mm diameter) on top.

### Recording sessions

Departing and approach flights were recorded with a high-speed digital stereo camera system. Two synchronised video cameras (Redlake MotionPro500) where positioned at a distance of about 2m above the arena and allowed us to measure the position and orientation of the body length axis at 250 frames/sec with a resolution of 1024x1024 pixels in each view. The optical axis of one of the two stereo cameras was levelled with respect to gravity and pointed straight down; the second camera covered roughly the same visual field, of about 1m^2^ around the landmark arrangement from a slightly different angle. Video sequences were stored as uncompressed 8-Bit image files in tiff format on computer hard disk for off-line processing. With these parameter settings the maximum recording time was restricted by the onboard memory of our video cameras to 16s.

### Data Analysis

The position of the bee and the orientation of its body length axis were automatically determined in each video frame by custom-built software (https://opensource.cit-ec.de/projects/ivtools) for both image sequences provided by the stereo video camera system. We determined the bee’s body yaw angle from the levelled camera. The three-dimensional position coordinates were then computed by using both camera views. For camera calibration and 3D stereo triangulation we used the Camera Calibration Toolbox for Matlab by Bouguet [39]. 3D coordinates and the yaw body orientation of the bee were then low-pass filtered (second-order Butterworth filter) with a cut-off frequency of 20Hz.

We also used our custom-built computer program to measure the bee’s head position and yaw orientation in the image sequences. The centre of the bee’s head was manually marked by clicking on it in every frame of the sequence. A new image was then generated taking a 90 by 90 pixel sized region around the centre of the head. The image was manually rotated using the computer mouse until it reached the vertical reference direction. The negative value of the angle, which was used to straighten the image, then indicated the yaw orientation of the bee’s head relative to the orientation of the camera. This method is illustrated in S1 File. Orientation measurements were greatly facilitated by this method and tracking errors were easy to detect this way. We checked the precision of our methods by analysing the differences of orientation measurements that were done by three different observers in a given image sequence. These differences were on average smaller than 1°. We also compared manual and automatic measurements of the bee’s body orientation and found that differences were also smaller than 1°. All head data are available from the Bielefeld University data archive at http://doi.org/10.4119/unibi/2763686.

For detecting saccades we used a yaw angular velocity threshold criterion and a criterion that was sensitive to the direction of movement; this procedure was derived from the method used in [40]. Only if the absolute value of saccade velocity exceeded 200°/sec, and the head moved in the same direction for at least five frames (20ms in total) was a turn classified as a saccade. Once head saccades were detected this way, we determined the maximum angular velocity and went 70ms back and 70ms forth in time to also capture the rising and falling phase of the angular velocity. We then searched again for the start and end points of every single saccade as defined by the yaw angular velocity threshold criterion (>200°/sec) and a duration criterion (same direction for at least four frames). These operations, the statistical testing and all further calculations, e.g. the quantification of saccade amplitudes, velocities and durations were done in Matlab (R2010a, The Mathworks, Natick, MA, USA).

To compare samples from two or more groups we used the Kruskal-Wallis test as implemented in the Matlab Statistics Toolbox (Version 7.3). It returns the p value for the null hypothesis that all samples are drawn from the same population. In case of significant effects we used Tukey's honestly significant difference criterion for post-hoc multiple comparisons to find out which data groups are significantly different from each other.

## Results

After some training bees travelled regularly through the plastic tubing between hive box and flight arena. They entered the arena via a hole in the sidewall from where they flew towards the feeder (F in Fig 1A). We measured the time needed to land on the feeder after entering the flight arena in four different bumblebees (33 flights). Trained bumblebees needed only about ten seconds to find the feeder in this experiment, demonstrating their extraordinary homing capabilities. For comparison we plot honeybee search times (14 different honeybees; 68 flights; taken from [10]), which seem to take a little longer to find the feeder, most likely because we focussed on a few highly trained bumblebees in the present study (Fig 1B). To test whether the bumblebees really learned the position of the feeder relative to the three landmarks, or rather relied on additional navigational mechanisms like path integration, we changed the position of the landmark arrangement within the arena, while maintaining the geometry of the landmark arrangement (n = 74 flights) constant. This did not drastically change the search times (Fig 1C). Even though search times for the two alternative locations are significantly different compared to the main training and filming site (p = 0.02), they are well within the range of honeybee search times. Even though bees had to fly a much shorter path to one of the two alternative feeder locations the search time for the two alternative locations are not significantly different from each other, (p = 0.8). It is therefore unlikely that the bees were predominantly using path integration or other visual cues available in the circular flight arena to find the feeder and mostly relied on using the three cylinders as landmarks.

**Fig 2:**
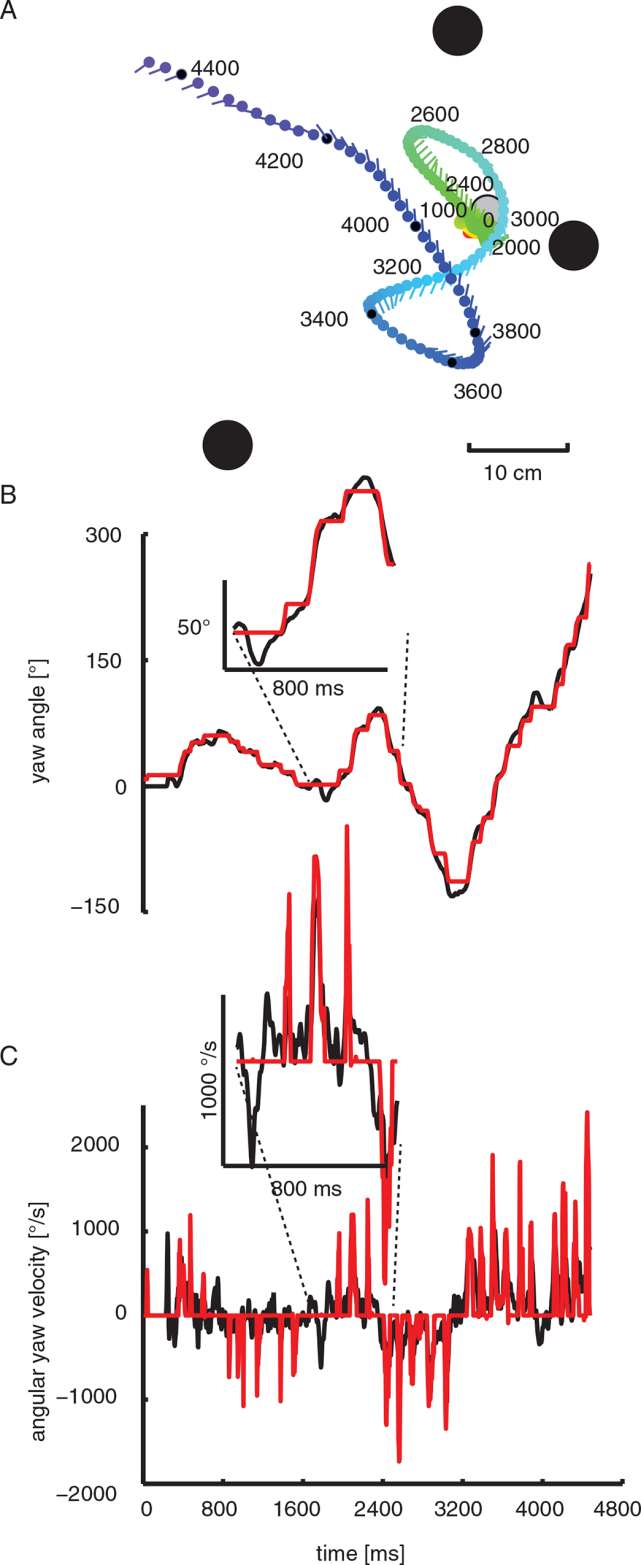
Example of turn-back-and-look flight (TBL). (A) Top view of the typical flight trajectory of a bumblebee departing from the feeder (light grey circle). The position of the bee’s head is shown every 16 ms. During the initial sections of this "turnback- and-look” (TBL) flight the bee is facing the goal while backing away from it. The three landmarks are drawn in dark grey. (B) Head yaw angle (red) and body yaw angle (black) for the flight shown in A. It illustrates that the bee’s head orientation (black) can deviate considerably from the yaw orientation of its body. The head usually turns with the thorax but at a higher angular speed, starting and finishing slightly earlier. (C) Head (red) and body (black) yaw angular velocity for the same flight. Head saccades partially coincide with body saccades, but not each body saccade leads to a head saccade. Inset: magnification of the yaw velocities illustrating the coincidence of saccadic head and body saccades.

The bees often performed learning flights at the entrance to the flight arena as well at the feeder position during departure. This was especially prominent when we had changed the position or the appearance of the feeder. These learning flights presumably enable bees to store and reacquire similar nest-focused views during learning and return flights [12;41]. We recorded the ‘turn-back-and-look’ (TBL) learning flights of bees leaving the feeder on the first few times (Fig 2A) in the vicinity of the landmark arrangement. To assess the visual input bumblebees perceive and to relate commonalities between learning and return flights that might improve the bees’ local navigation performance we analysed recordings of 6 learning flights and the following 19 return flights in detail. During these flights, the bee’s body yaw direction is kept nearly constant except for brief periods when yaw orientation changes quickly (Fig 2B). These yaw body turns (‘body saccades’) are often accompanied by even faster head yaw turns (‘head saccades’). The body saccade starts slightly earlier compared to the head saccade and also ends later compared to the shorter head saccades. The time course of body and head yaw orientation is very similar, with the difference that the head orientation is more constant than body yaw orientation between saccades and angle changes of the head are performed in a more step-like manner. Maximum yaw velocities are therefore higher for the head than for the body, since the head orientation stays largely constant for longer durations between saccades, than does the body between its turns (Fig 2C). This flight pattern of bumblebees is very similar to that of honeybees (Boeddeker et al. 2010). Since the bee’s head position and orientation determine the visual input, we will focus only on head coordinates in the following.

**Fig 3:**
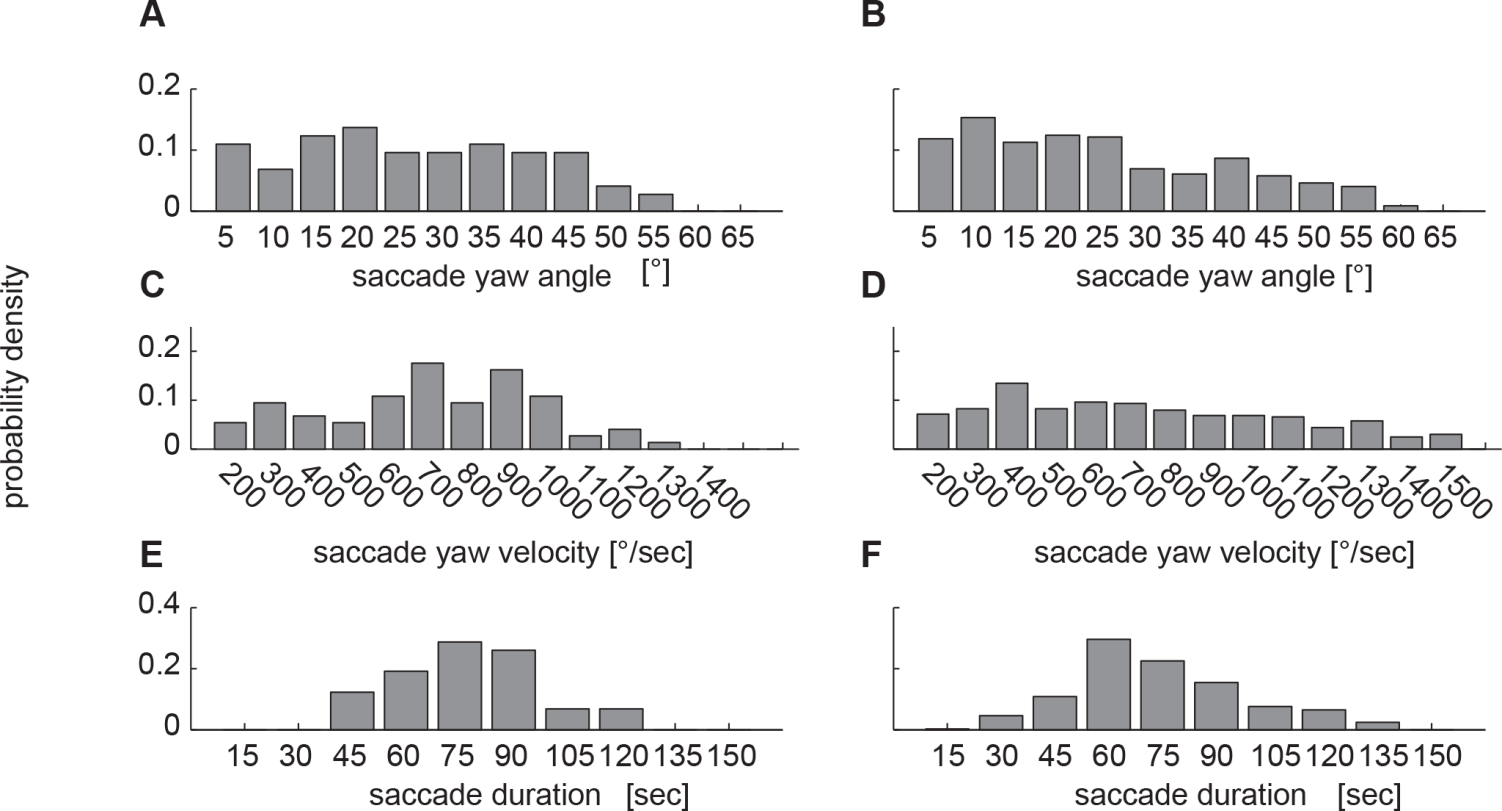
Angular velocity and amplitude distributions. Histograms for the amplitude, velocity and duration of head saccades are calculated from a total of 443 saccadic head movements for different head saccade size classes from six different bumblebees. Saccades were detected as peaks in yaw angular velocity. Each data plot is normalized to sum up to one. (A, C, E) TBL flight saccade size, saccade velocity and saccade duration (N = 6, n = 74). (B, D, F) size, velocity and duration distributions for return flights (N = 6, n = 443).

We divided the data of learning (TBL) and return flights into the two characteristic phases: ‘saccades’, when angular velocities of the head reach up to 1500°/sec (see Fig 2C), and ‘intersaccades’, when the yaw orientation of the head is kept virtually constant. To compare the characteristics of head saccades during learning and return flights, we calculated histograms of the amplitudes, velocities and durations of head saccades (Fig 3). It turned out that these are fairly similar for the two types of flights, with broad distributions of head yaw angles between 5° and about 60° (Fig 3A and 3B), but also—to a lesser degree—of saccade duration that typically last for between 45ms to 135ms (Fig 3E and 3F). Learning and return flights differed only slightly in their saccade yaw velocities: Saccades with high angular velocity appear to occur slightly more often during return flights (Fig 3C and 3D). This could be due to the stereotypical flight choreography of learning flights, where the animal moves in loops and arcs in short straight flight segments during which gaze direction is kept constant. These segments are linked by saccadic head movements against the direction of the pivoting arc around the goal location. The saccadic head movements during learning flights seem to be part of an inherent motor programme that is present and very similar in all flying hymenopteran insects that have been analysed in detail (e.g. [13; 20]). The occurrence of larger saccades during return flights than during learning flights might facilitate faster scanning of a larger part of the surroundings of the goal. Saccades during search flights appear to be distributed less regularly and less stereotypical in their sequence, although they also share many similarities or “motifs” with learning flights [42].

**Fig 4:**
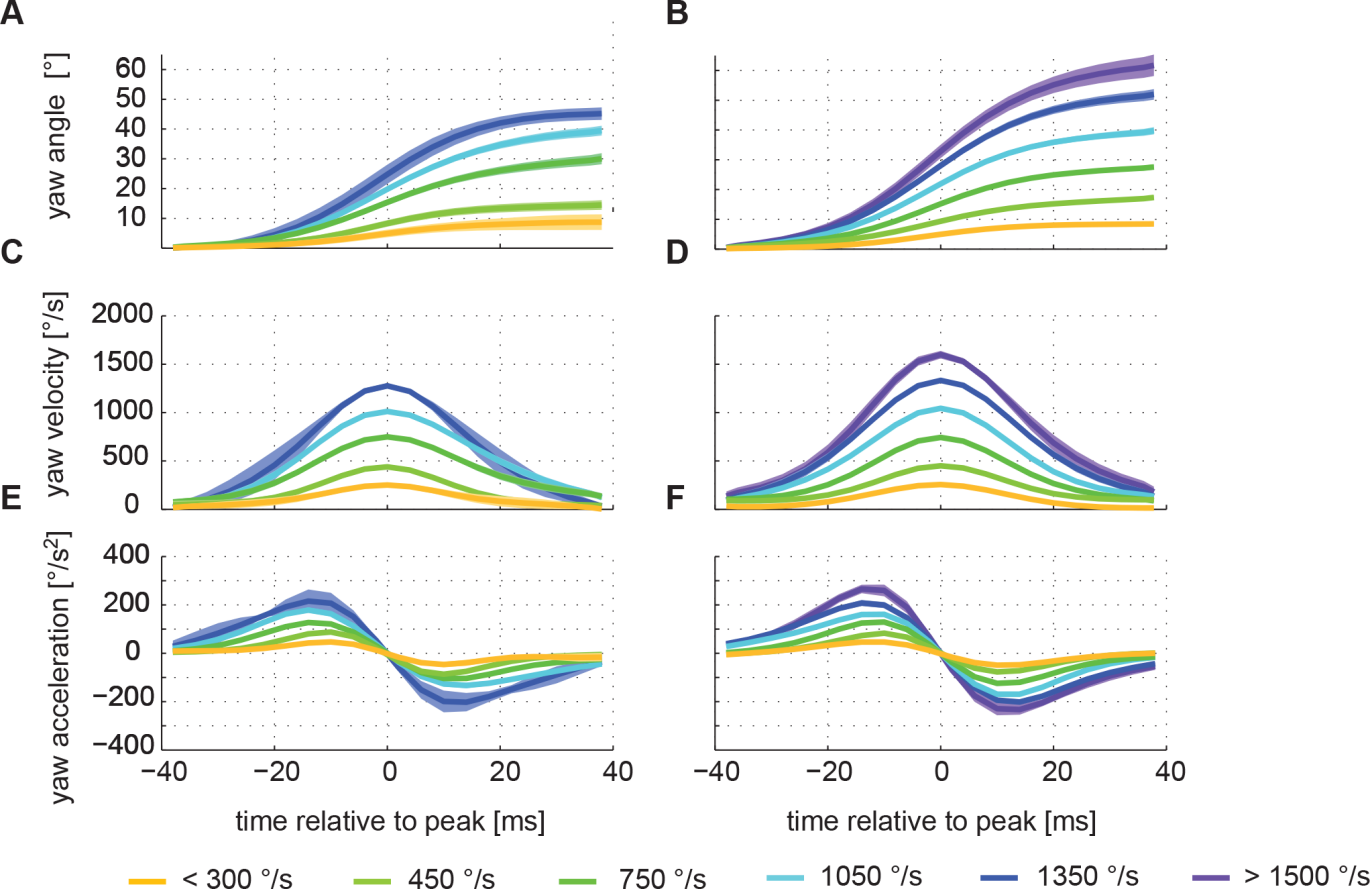
Average saccade amplitude, velocity and acceleration profiles. Saccade-triggered averages of head yaw orientation, velocity, and acceleration for all TBL (A, C, E; N = 6, n = 74) and return flights (B, D, F; N = 6, n = 443). The means were calculated from all saccades that fall within one of the velocity classes (see legend). Data are centred on the peak velocity of the respective saccade. All but the smallest and the largest velocity classes have a width of 300°/s and the numbers give the mean of the respective class. The shaded areas around the average lines (mean) depict the standard error of the mean. The angular velocity profile of saccadic head movements is very similar for different head saccade size classes and also for the two types of flights.

**Fig 5:**
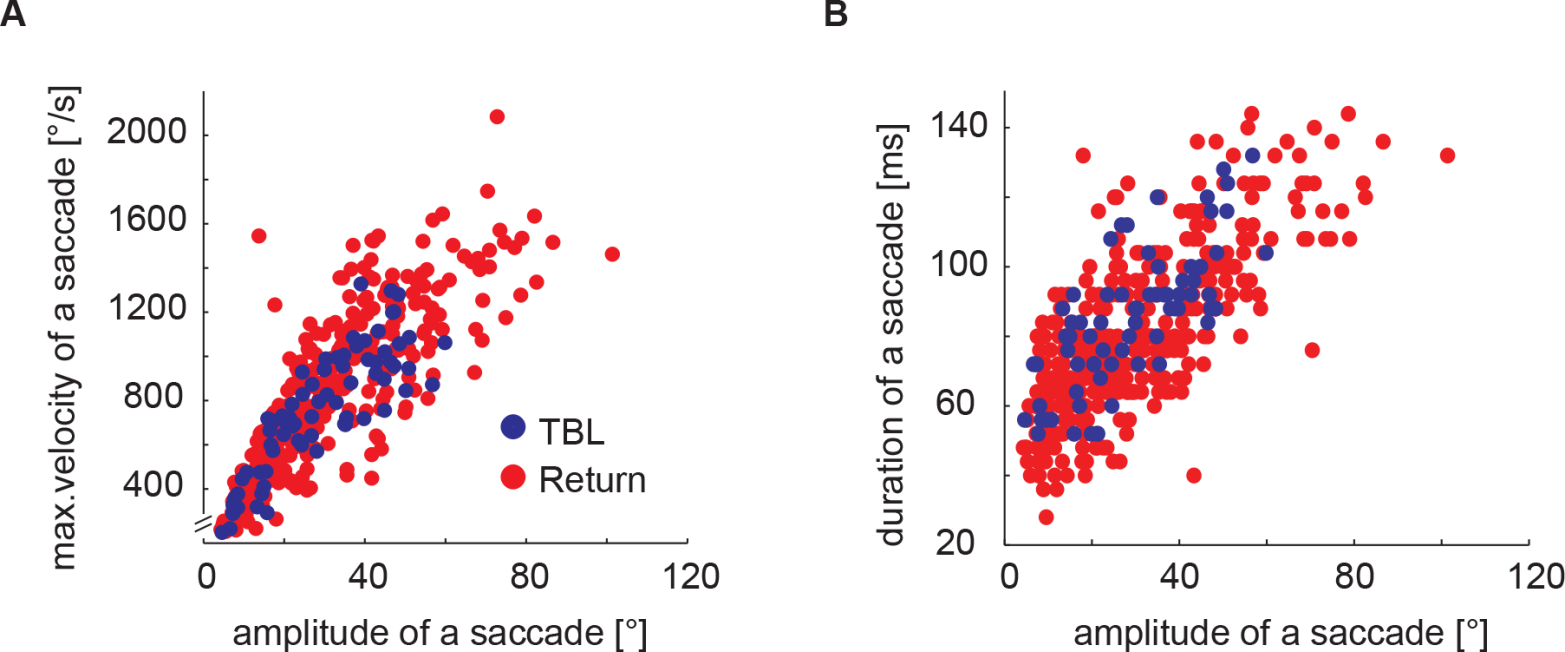
Correlation between duration, peak velocity and amplitude of saccadic head movements. The saccade duration was calculated from the start and end points as determined with the saccade finding algorithm that uses a combined velocity threshold and slope criterion (see results section). Saccades from the two kinds of flight both follow a strict pattern and show very similar characteristics on the fine time scale analysed here. For this reason we take the two types together for the correlation analysis (N = 6, n = 517). (A) shows a strong relationship between saccade amplitude and velocity (Pearson's r = 0.84) and (B) also demonstrates that saccades are highly stereotypical movements with the duration and saccade amplitude being closely related (r = 0.74). Please note that the regular pattern visible in (B) is due to the sampling interval of our high-speed video system (4ms).

Resolving saccades at a finer time scale we see that all saccades show a similar pattern following a stereotyped time course that does not much change with saccade velocity (Fig 4). Although the distribution of measured saccade parameters is quite broad with saccade peak velocities ranging from below 250 to 1500°/sec, we find many similarities between the differently sized saccades. The shape of the angular velocity profile of saccadic head movements is alike for different saccade sizes (Fig 4A and 4B) with an apparently symmetrical rise and fall of the angular velocity (Fig 4C and 4D). This is better visible in the yaw acceleration time profiles (Fig 4E and 4F). Most of these time profiles are symmetrical with respect to the maximum yaw velocity (0ms). Only the profiles of the two largest saccade amplitude classes (1200°/s– 1500°/s and < 1500°/s, which are only present during return flights) are significantly asymmetrical. For quantification of this asymmetry we took the integral of the first half of the acceleration profile (from -38ms to 0ms) and subtracted the integral of the second half of this profile for every saccade (0ms to 38ms). This value will differ from zero if the rising and the falling edges are asymmetrical. Only the two largest saccade classes proofed to be significantly different from zero difference in this measure (p < 0.01), whereas all other classes are symmetrical. Thus, saccades within the most common velocity classes between 100°/sec and 1350°/s share a very similar angular velocity profile. To check whether saccades with different amplitudes also have a similar width, we fitted a Gaussian velocity profile to every saccade using the ‘fminsearch’ function in Matlab. The fitting parameters were the location of the mean, sigma (standard deviation), the offset value, and the gain value). We find that the scaling (gain) is indeed the main factor that determines the difference between small and large saccades since saccade width is similar for all saccades (mean sigma 12.8 ms ± 1ms) independent of their size (p = 0.2682). Another characteristic feature of bumblebee head saccades is the tight relationship between the velocity with which the head moves and the saccade amplitude. Saccade amplitude and velocity are highly correlated (Fig 5A; r = 0.84). Even though the underlying motor programme might be very similar for the differently sized saccades, the durations are different. This might seem slightly paradoxical in the first place, but is mainly a consequence of the threshold criterion we use to detect the onset and the end of a saccade (see above). The saccade-defining threshold is reached earlier due to the higher velocity of large saccades compared to small saccades. Saccade duration thus varies between 30ms for the smallest to over 100ms (Fig 3E and 3F; Fig 5B). The correlation between saccade duration and saccade amplitude (Fig 5B; r = 0.74) further indicates that saccades follow a highly stereotypical pattern.

## Discussion

Bumblebees change gaze direction by short and fast head yaw turns, reducing image rotation to brief time intervals. Why is it so important to control gaze orientation? We assume that facilitation of depth perception from motion parallax is one important reason, because visual mechanisms that exploit the translational components of optic flow for odometry or depth perception are likely to break down, if contaminated by strong rotational optic flow. The detailed analysis of the fine structure of the bees’ head turning movements shows that the time course of single head saccades is very stereotypical.

### Which sensory cues do bees exploit to orient their head and how are head and body movements coordinated?

A recent study shows that flying honeybees stabilise head roll orientation visually [24], and that vision dominantly controls head roll rotations. However, many details of the mechanisms controlling the bees’ head and body rotations remain to be determined. Also the question of what other sensory and neuronal mechanisms might assist in stabilising gaze against roll and yaw rotations during flight remains unsolved. A possible role may be attributed to hard-wired motor programs that might assist head-body coordination in both bees and flies. In humans, similar feed-forward models were proposed to predict the sensory consequences of actions and are thought to play a crucial role for understanding motor control [43]. Moreover, there is recent evidence from some insect species for the predictive modulation of sensory processes by motor output [44–45]. Information on saccade timing and rotational optic flow might be conveyed to neck muscles to stabilize head orientation except for the brief periods of saccadic head orientation changes.

### What is the functional advantage of the stereotypical eye movements?

The kinematics of the differently sized saccades seems to be determined by a tightly controlled and stereotypical motor programme. Saccadic head movements in bumblebees share many similarities with saccades in other insects and even in humans and monkeys. The consistent relationship between the duration, peak velocity and amplitude of human saccadic eye movements is known as the 'main sequence' [46]. The reason why such a stereotypical relationships evolved is unknown [47]. It was shown that the stereotypical durations and trajectories are optimal for minimizing the variability in saccade endpoints caused by motor noise [48], which might also be relevant for bumblebee saccades.

### What is the impact of morphological differences between species and within species on vision and flight performance?

Head saccades in bees with their maximal yaw velocities around 1500°/s are slower than head saccades in flies where yaw velocities above 2500°/s have been measured [26]. Experiments by Hengstenberg [49] and Sherman & Dickinson [50–51] show that the fly’s visual system is tuned to relatively slow rotation, whereas the haltere-mediated response to mechanical rotation increases with increasing angular velocity. Up to now it is not yet clear, whether honeybees or bumblebees possess specialised inertial sensors. The advantage of specialised inertial sensors over visual feedback is their much shorter response delay. The latency measured in neck motor neurones from haltere deflection is only about 3ms in blowflies [52], whereas visual motion stimuli evoke neural activity in the brain of flies with a delay of about 20–30ms [53]. These findings indicate that fast haltere-mediated reflexes help flies to control their fast head-body coordination and thus enable them to perform very rapid flight manoeuvres. Rapid flight manoeuvres are the harder to perform the larger the animal’s body weight is, and the further the centre of mass is shifted away from the wingbases, which increases the moment of inertia [54]. As the bumblebees’ centre of mass is shifted strongly to the abdomen, these restrictions make it even more important for the bumblebee to cancel out rotational optic flow via head-body coordination.

Individual bumblebees also differ largely in size, which has certain implications for their visual system. For instance, larger bumblebees have been shown to be more precise in single target detection [55] than smaller specimen. The same study revealed that the number of ommatidia involved in object detection correlates with body size. Since we did not want to address the complex issue of a potential size dependence of the fine structure of homing flights, we used only medium-sized bumblebees in our study. Even between the closely related honeybees and bumblebees a number of differences could be found that might influence their navigational performance. Compared to honeybees bumblebees possess an approximately 25% higher spatial resolution than honeybees since they can resolve gratings with higher spatial frequencies, indicating a larger visual acuity [56–57]. And even though both, honeybees and bumblebees, have almost identical photoreceptor sensitivity spectra [58], colour discrimination performance of bumblebees is not as good as that of honeybees [59]. Despite these differences in the visual system we found honeybees and bumblebees to exhibit a very similar performance in navigation according to visual landmarks.

### Reasons for a partial decoupling of head and body orientation

We find here that in bumblebees (Fig 3C) the general relationship between head and body orientation is very similar to flies and honeybees [24–26]. Related studies on bumblebees come to somewhat different results and conclusions. Hempel de Ibarra et al. [23] and Philippides et al. [42] conclude that the bumblebees’ body orientation allows a reasonable estimate of gaze direction without measuring head orientation. However, they also report an average 5–6° deviation of the head orientation compared to body orientation, which is consentient with the data reported here. The apparent contradiction can be attributed to the temporal scale of the behavioural analysis. A more recent study by Riabinina et al. [60] finds–similar to our present study–fast head saccades during which head and body orientation deviate significantly. But, contrary to our study, Riabinina and colleges [61] find that during fixations between saccades, the bumblebee’s head continues to turn slowly, generating slow rotational flow. The authors conclude that “at specific points in learning flights these imperfect fixations generate a form of ‘pivoting parallax’, which is centred on the nest and enhances the visibility of features near the nest” (from [60]; see also [61] for the concept of ‘pivoting parallax’). The bumblebee’s saccadic strategy may thus slightly differ from other reported cases. Instead of fully stabilising the head between saccades, bumblebees might not always move their heads enough to reduce rotational image speed completely. We also noticed slow rotations between saccades in our flight sequences, but only in less than 10% of flight time. In the present study we decided to not analyse these parts of flights since we could not exclude methodological noise as a source. It remains a topic for future high-resolution behavioural studies to analyse in greater detail what function might be driving slow head rotations during learning flights. One other possibility to assess the function of slow rotations during learning flights might be to investigate their impact on motion sensing neurones. For blowflies, Kern and colleagues could show that fine temporal differences in head and body rotations of blowflies turn out to be relevant for motion processing in the fly’s visual system [27]. More specifically, they revealed that if the fly’s head were tightly coupled to the body, the resulting optic flow during intersaccades would not contain behaviourally relevant information about the spatial layout of the environment. In a similar way, we recently showed that landmark features are indeed represented in the bee’s visual motion pathway [34]. These neurons convey information about landmark properties that are useful for view-based homing. It is quite likely that losing this information would heavily impair the bees’ ability to navigate on a local scale using visual landmarks via optic flow as a relevant cue helping to find the goal location [10; 62]. Whether and to what extent motion processing in the bumblebee’s visual system is adapted to their flight style and how well bees might be able to distinguish one spatial setting from others remains to be tested in electrophysiological and further behavioural experiments. Since the dynamics of the visual input perceived by a homing bee crucially depend on the dynamics of its behaviour, the detailed knowledge on the behavioural dynamics of eye movements as presented here, provides the basis for these future experiments on the coding properties of visual motion sensitive neurones in the bumblebee brain.

## Supporting Information

S1 File Close-up with overlaid tracked angles onto an original movie. This movie illustrates the methods used in this paper to determine head orientation in bumblebees. The centre of the bee’s head was manually marked by clicking on it in every frame of the sequence. A new image was then generated taking a 90 by 90 pixel sized region around the centre of the head. The image was manually rotated using the computer mouse until it reached the vertical reference direction. The negative value of the angle, which was used to straighten the image, then indicated the yaw orientation of the bee’s head relative to the orientation of the camera.
(MOV)
